# Association between perinatal hypoxic-ischemia and periventricular leukomalacia in preterm infants: A systematic review and meta-analysis

**DOI:** 10.1371/journal.pone.0184993

**Published:** 2017-09-20

**Authors:** Jichong Huang, Li Zhang, Bingyao Kang, Tingting Zhu, Yafei Li, Fengyan Zhao, Yi Qu, Dezhi Mu

**Affiliations:** 1 Department of Pediatrics, West China Second University Hospital, Sichuan University, Chengdu, China; 2 Key Laboratory of Obstetric & Gynecologic and Pediatric Diseases and Birth Defects of Ministry of Education, Sichuan University, Chengdu, China; Shanghai Jiaotong University School of Medicine Xinhua Hospital, CHINA

## Abstract

**Background:**

Although investigators have implicated hypoxic-ischemia (HI) as a potential cause of periventricular leukomalacia (PVL), the role of clinical risk factors or markers for HI in the development of PVL remains controversial. The aim of this study was to identify perinatal HI-related factors associated with PVL.

**Method:**

The PubMed, EMBASE, and Cochrane Library databases were searched. The last search was performed on January 2017. Summary effect estimates (pooled odds ratios [ORs]) were calculated for each risk factor using fixed or random effects models with tests for heterogeneity and publication bias.

**Results:**

Fifteen studies with a total of 12,851 participants were included in this meta-analysis, and 14 potential risk factors were analyzed. The pooled results showed that mothers with oligohydramnios (OR, 1.55; 95% confidence interval [CI], 1.05 to 2.30), preterm infants with acidemia (OR, 1.87; 95% CI, 1.18 to 2.97), 1-minute Apgar score <7 (OR 2.69; 95% CI, 1.13 to 6.41), 5-minute Apgar score <7 (OR, 1.89; 95% CI, 1.39 to 2.56), apnea (OR, 1.76; 95% CI, 1.07 to 2.90), respiratory distress syndrome (OR, 1.46; 95% CI, 1.04 to 2.03), and seizures (OR, 4.60; 95% CI, 2.84 to 7.46) were associated with increased risk of PVL.

**Conclusion:**

This study identified perinatal HI-related risk factors for the development of PVL in preterm infants. Future large-scale prospective clinical studies are required to validate and extend these findings.

## Introduction

Periventricular leukomalacia (PVL) is the most common form of cerebral white matter injury in preterm infants and results in cerebral palsy in 60–100% of survivors [1, 2]. The majority of the theories consider the necrotic foci in white matter to be hypoxic-ischemic lesions [3]. Periventricular white matter in preterm infants is extremely sensitive to perfusion-related injury, as it receives only 25% of the blood flow that the cortical gray matter receives through short or long penetrating arteries. Moreover, the neurovasculature of the white matter in preterm infants is particularly immature and therefore shows a poor ability to self-regulate blood flow [4, 5]. Therefore, factors related to perinatal hypoxic-ischemia (HI) may play an important role in the development of PVL in preterm infants.

There are several perinatal HI-related risk factors implicated in numerous retrospective studies that may be related to PVL [6–9]. These include maternal and placental problems (e.g., preeclampsia, oligohydramnios, and abruption placenta), and fetal and neonatal disorders (e.g., acidemia, low Apgar score, respiratory distress syndrome, and seizures). However, the overall conclusions of these studies are inconsistent. For example, preeclampsia as an important maternal complication that reduces placental blood perfusion has been implicated in many studies that have analyzed its association with PVL. However, no investigators have found significant results [9–11]. In addition, some studies have found that neonatal seizures increase the risk of PVL [6, 12, 13], while others have reported that there is no significant difference between cases and controls in terms of seizures [9, 10, 14]. Therefore, it is necessary to determine the influence of clinical risk factors or markers for HI in the development of PVL in preterm infants. We thus carried out a systematic review and meta-analysis to determine the relationships between HI-related factors and PVL.

## Materials and methods

### Study identification and selection

The study was conducted according to the Preferred Reporting Items for Systematic Reviews and Meta-Analyses criteria (PRISMA) as shown in S1 File. The PubMed, EMBASE, and Cochrane Library databases were searched using the following keywords and subject terms: “periventricular leukomalacia” OR “periventricular atrophy” OR “periventricular white matter abnormalities” OR “white matter injury” OR “white matter damage” OR “white matter infarct” OR “white matter abnormality” OR “white matter reduction” AND “risk factor.” The last search was performed on January 2017. The search was limited to human studies without language restriction. The titles and abstracts of all studies were initially screened and the full texts of potential studies were then read independently by two of the authors (Yafei Li and Fengyan Zhao) of this study to determine their eligibility according to the following inclusion criteria: (a) those that investigated the association between perinatal HI-related factors and PVL; (b) case-control or cohort studies; and (c) those that reported risk ratios (RRs) or odds ratios (ORs) and corresponding 95% confidence intervals (CIs) or other available data to calculate the CIs. The following exclusion criteria were used: (a) studies with overlapped populations; (b) those that did not include a control group; and (c) those that provided unavailable original data, such as studies that expressed Apgar score as means and standard deviations. Any disagreements were reconciled by a third author (Jichong Huang), who independently reviewed the studies and then discussed them with the initial two reviewers until a consensus was reached.

### Data extraction

The literature on PVL was scrutinized in detail by two authors (Yafei Li and Fengyan Zhao) to identify all possible risk factors which might cause hypoxia ischemic injury. The two authors independently extracted the following data from each study included in the meta-analysis: first author, publication year, country of origin, study design, sample size, gestational age, birth weight, assessment methods for PVL and exposure, and risk of bias. The ORs and 95% CIs were extracted from the studies or computed by authors using Review Manager 5.3 software if they were not provided. After data extraction was completed, the results were compared by two authors. Any disagreement was resolved by a third author (Jichong Huang).

### Quality evaluation

The methodological quality of each included study was independently examined by two authors (Bingyao Kang and Tingting Zhu) using the Newcastle-Ottawa Scale (NOS) [15]. The quality scores ranged from 0 to 9. Quality was determined based on group selection (four items), comparability between groups (one item), and outcome and exposure assessment (three items). Studies with scores of at least 5 were considered high-quality studies [16]. Disagreements between the two reviewing authors were examined by a third author (Jichong Huang) and then discussed until a consensus was reached.

### Statistical analysis

We reviewed the possible perinatal HI-related risk factors for PVL, which included prepartum complications, intrapartum complications, and infant characteristics. We only analyzed risk factors that were evaluated in two or more studies. A separate meta-analysis was conducted for each risk factor and the pooled OR was calculated using a fixed-effect model or a random-effect model. A fixed-effect model was used when heterogeneity across studies was not detected (a P-value >0.05 indicated heterogeneity). Otherwise, a random-effect model was used [17, 18]. Heterogeneity among studies was assessed using Chi^2^ statistics and I^2^ tests. Data were considered statistically heterogeneous if I^2^ was >50% and the P-value was <0.05. Forest plots were used to show ORs and 95% CIs for each individual study, as well as pooled ORs and 95% CIs. Publication bias was evaluated using funnel plots and the Begg’s test [19], wherein a P-value <0.05 was considered statistically significant. All analyses were performed using Review Manager 5.3 and Stata 12.0 software.

## Results

### Study selection process and characteristics

**Fig 1:**
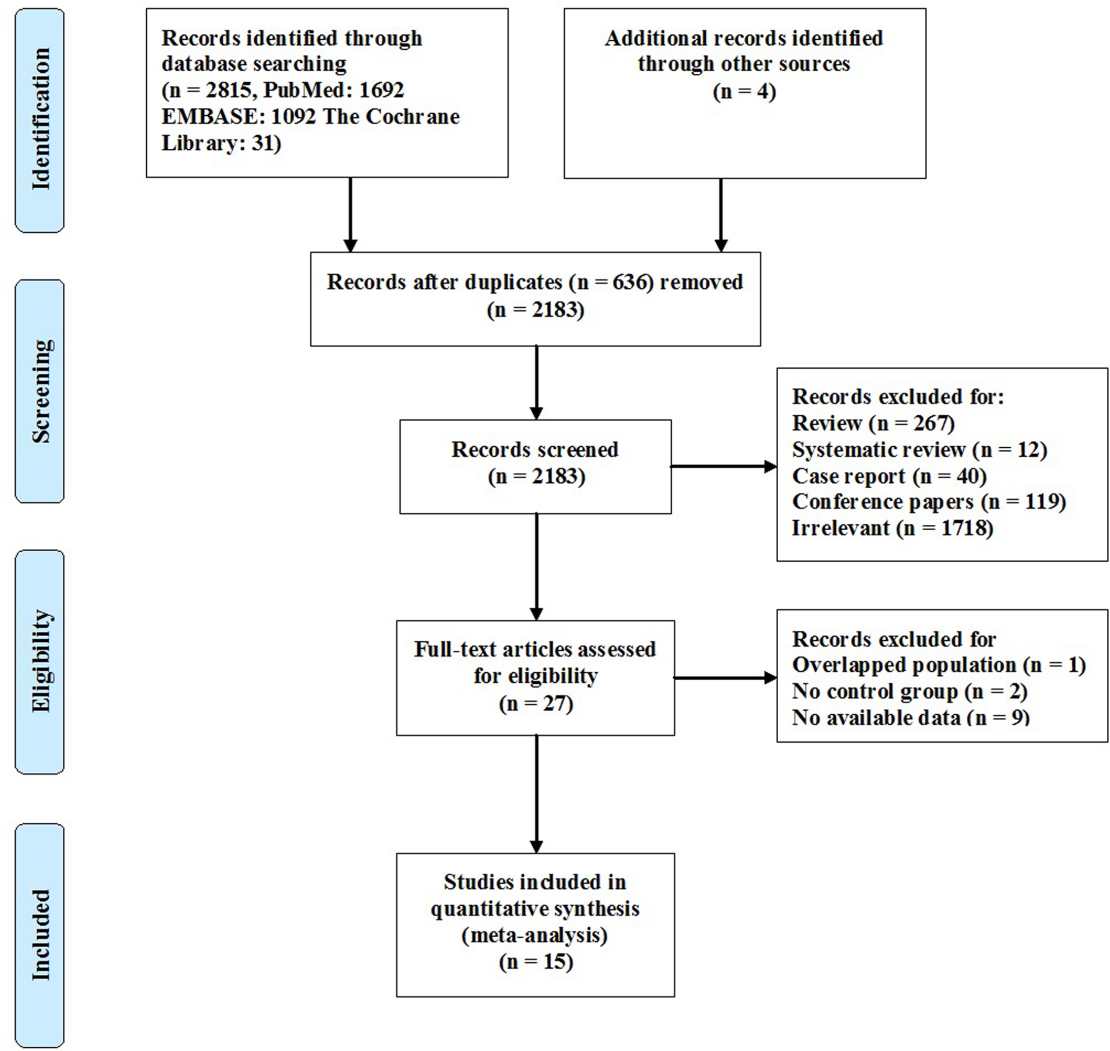
Flow diagram of the study selection process.

A total of 2,183 studies, excluding duplicate publications, were identified by our search strategy. Twenty-seven potential studies were eligible for further full-text review after the screening of titles and abstracts. After reading the full texts, 1 study was excluded for overlapped populations, 2 studies were excluded for not having a control group, and 9 studies were excluded for unavailable data. Fifteen studies were ultimately included in this meta-analysis. A flow diagram detailing the selection process is shown in Fig 1.

The included studies were published between 1996 and 2016. The sample sizes varied from 30 to 3,187. All of the studies involved preterm neonates (gestational age younger than 35 weeks). All of the studies used cranial ultrasound for the assessment of PVL, but the timing of assessment varied from 72 hours to 40 weeks after birth. A summary of the characteristics of each included study is presented in Table 1.

**Table 1:** Characteristics of the included studies.

Author, year	Country	Study design	Size		GA(w)	BW(g)	Ascertainment of exposure; outcome	Risk of bias; quality
Tsimis 2016 [13]	United States	Retrospective cohort	374	case:26.3±2.2 control:28.0±2.5		case:868±237 control:993±276	Medical records; cranial ultrasound 6 weeks after birth	No representativeness of the exposed cohort;NOS:8
Tawil 2012 [6]	Kingdom of Saudi Arabia	Case-control	118	case:27.4 control:26.2		case:1019.0 control:824.3	Medical records; cranial ultrasound at 1 week, 2 weeks and 5–6 weeks after birth	No representativeness of the cases, no report of non-response rate;NOS:7
Resch 2012 [14]	Austria	Case-control	141	case:30.3±2.3(25–35) control:30.4±2.4(24–35)		case:1450±550(618–2500) control:1446±358(685–2510)	Medical records; cranial ultrasound at days 1, 3, and 5 of life and repeated weekly until diagnosis	No representativeness of the cases, no report of non-response rate;NOS:7
Hatzidaki 2009 [12]	Greece	Case-control	135	case:30.1±2.6;control:30.4±2.4		case:1278.4±428.2 control:1375.5±378.6	Medical records; cranial ultrasound within 1 week after birth	No representativeness of the cases, no report of non-response rate;NOS:7
Bauer 2009 [11]	Austria	Case-control	340	case:31.1±2.5 control:31.3±2.5		case:1278.4±428.2 control:1518±425	Medical records; cranial ultrasound within 7 weeks after birth	No representativeness of the cases, no report of non-response rate;NOS:7
Oda 2008 [20]	Japan	Retrospective cohort	30	case:28.7±1.94 control:30.0±2.37		case:1410±402 control:1525±525	Medical records; cranial ultrasound before 72 hours and at 2 weeks after birth	No representativeness of the exposed cohort, follow-up was not long enough for outcome;NOS:7
Silva 2006 [10]	United States	Case-control	352	case:27.3±2.7 control:27.4±2.7		case:958±306 control:1038±381	Medical records; cranial ultrasound	No representativeness of the cases, no report of non-response rate;NOS:7
Murata 2005 [8]	Japan	Retrospective cohort	201	case:29.4±2.1 control:29.6±2.1		case:1324±324 control:1304±371	Medical records; cranial ultrasound within 4 weeks after birth	No representativeness of the exposed cohort, follow-up was not long enough for outcome;NOS:7
Locatelli 2005 [21]	Italy	Retrospective cohort	196	case:28.6±2.6 control:30.3±2.5		case:1183±362 control:1502±489	Medical records; cranial ultrasound at days 1, 3, and 7 of life and repeated every 2 weeks until 40 postconceptional weeks	No representativeness of the exposed cohort;NOS:8
Graham 2004 [9]	United States	Case-control	150	case:22.5±2.7 control:27.4±2.6		case:1053±402 control:966±285	Medical records; cranial ultrasound 6 weeks after birth	No representativeness of the cases, no report of non-response rate;NOS:7
Pladys 2001 [22]	France	Retrospective cohort	46	case:29.4(27.4–31.3) control:29.3(27–31.7)		case:1285(800–1945) control:1310(665–1980)	Medical records; cranial ultrasound within 6–8 weeks after birth	No representativeness of the exposed cohort, follow-up was not long enough for outcome;NOS:7
Kubota 2001 [7]	Japan	Case-control	51	29.8±2.7		case:1399±505 control:1410±479	Medical records; cranial ultrasound within 4 weeks after birth and once per week thereafter	No representativeness of the cases, no report of non-response rate;NOS:7
Resch 2000 [23]	Austria	Case-control	3187	31±2.5(26–35)		case:1511±456 control:1499±446	Medical records; cranial ultrasound at days 1, 3, and 5 of life and repeated weekly until diagnosis	No representativeness of the cases, no report of non-response rate;NOS:7
Spinillo 1998 [24]	Italy	Retrospective cohort	349	case:29.4±2.2 control:30.5±2.5		case:1267±378 control:1452±445	Medical records; cranial ultrasound within 2 weeks after birth	No representativeness of the exposed cohort;NOS:8
Perlman 1996 [25]	United States	Retrospective cohort	709	case:29.4±1.5 control:26.6±1.8		case:1285±301 control:904±248	Medical records; cranial ultrasound within 1 week after birth	No representativeness of the exposed cohort;NOS:8

Abbreviations: GA (w), gestational age (weeks); BW (g), birth weight (grams); NOS, score of Newcastle-Ottawa scale.

### Quality of included studies

The 15 studies included 8 case-control studies and 7 cohort studies. The methodological quality scores ranged from 7 to 8 points, and thus all studies were deemed to be of high quality. All studies used the medical records from one specific hospital or university rather than from a national health department, so the selection of cases in these studies may lack representativeness. Although all studies used cranial ultrasound as the method of diagnosis, the time of diagnosis varied between studies. Given that PVL can be diagnosed using a cranial ultrasound within 6 weeks of birth, the follow-up time was insufficient in three cohort studies [8, 20, 22]. None of the cohort studies provided a no-response rate at the end of follow-up.

### Perinatal HI-related risk factors

After scrutinizing the literature, we found 16 possible perinatal HI-related risk factors. Preterm premature rupture of membranes was excluded for its association with not only hypoxia-ischemia but also inflammation. Chronic lung disease (also known as bronchopulmonary dysplasia) was excluded for the inconsistency of the diagnostic criteria. Finally, 14 possible perinatal HI-related risk factors were identified (Table 2), including 6 prepartum or intrapartum complications and 8 infant characteristics. The statistically significant risk factors identified in the meta-analysis were oligohydramnios, acidemia, low Apgar score, apnea, respiratory distress syndrome (RDS), and seizures.

**Table 2:** Potential HI-related risk factors for PVL.

Risk factors	Numbers of studies	Pooled ORs (95% CI)	P-value*
**Prepartum/intrapartum risk factors**			
Preeclampsia/eclampsia	10	**0.68 (0.49, 0.93)**	**0.01**
Abruption placenta	7	1.19 (0.76, 1.87)	0.44
Oligohydramnios	4	**1,55 (1.05, 2.30)**	**0.03**
Intrauterine growth restriction	5	0.71 (0.33, 1.57)	0.40
Vaginal bleeding	4	1.30 (0.90, 1.88)	0.17
Breech delivery	3	0.87 (0.44, 1.74)	0.70
**Neonatal risk factors**			
Acidemia	6	**1.87 (1.18, 2.97)**	**<0.01**
Low Apgar score	7	**2.69 (1.13, 6.41)**^a^	**0.03**
		**1.89 (1.39, 2.56)**^b^	**<0.01**
Asphyxia	1	-	-
Apnea	2	**1.76 (1.07, 2.90)**	**0.03**
Respiratory distress syndrome	6	**1.46 (1.04, 2.03)**	**0.03**
Seizures	6	**4.60 (2.84, 7.46)**	**<0.01**
Patent ductus arteriosus requiring surgical treatment	4	1.22 (0.72, 2.05)	0.46
Hypocarbia	1	-	-

*: Significant positive result (*P* <0.05)

a: 1-minute Apgar score <7

b: 5-minute Apgar score <7

Abbreviations: OR, odds ratio; CI, confidence interval

### Oligohydramnios and PVL

**Fig 2:**
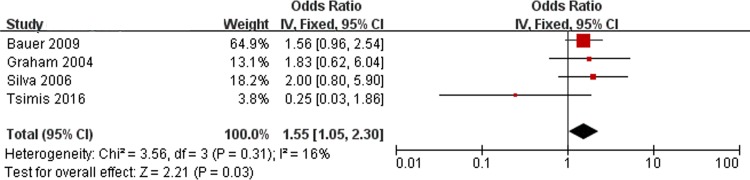
Fixed-effect model forest plot of the odds ratio of the association between oligohydramnios and PVL. The pooled OR of oligohydramnios is presented as a solid diamond at the bottom of the forest plot, the width of which represents the 95% CI.

Fig 2 shows the results of the combined ORs for oligohydramnios. There are 4 studies included in the meta-analysis. No significant heterogeneity was found (Chi^2^ = 3.56, *P* = 0.31, I^2^ = 16%). Thus, a fixed-effect model was used. There was a significant association between oligohydramnios and PVL (OR, 1.55; 95% CI, 1.05 to 2.30).

### Acidemia and PVL

**Fig 3:**
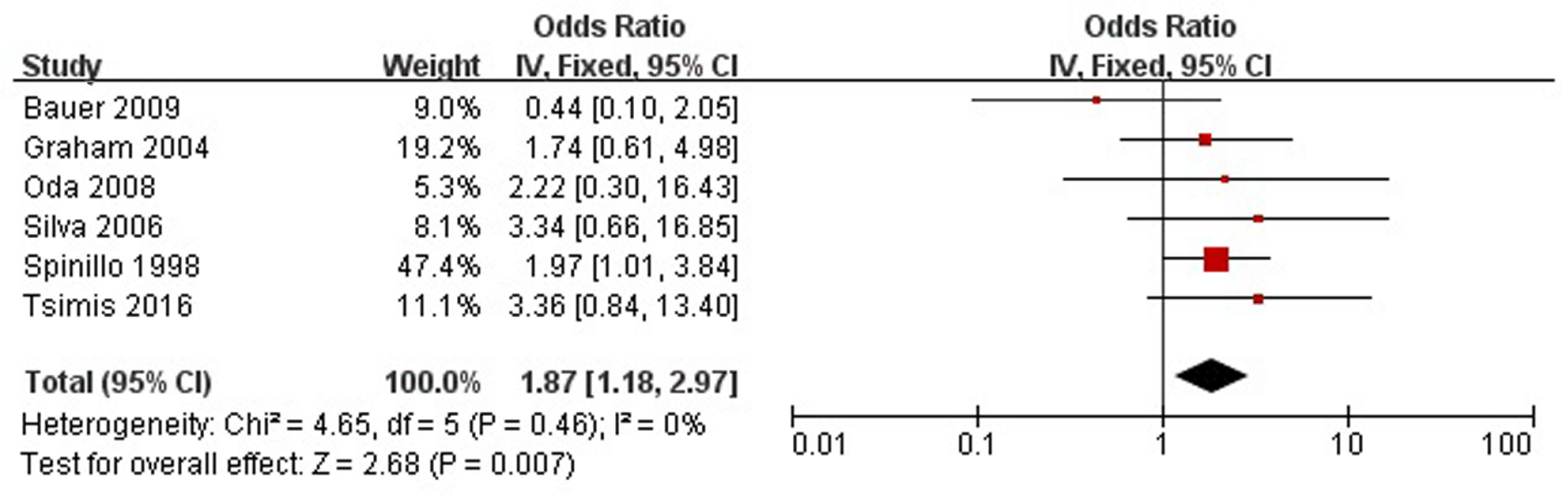
Fixed-effect model forest plot for the odds ratio of the association between acidemia and PVL. The pooled OR of acidemia is presented as a solid diamond at the bottom of the forest plot, the width of which represents the 95% CI.

Arterial umbilical cord pH <7.2 or base excess (BE) <-12.0 mmol/l was considered acidemia. There are 6 studies included in the meta-analysis (Fig 3). No significant heterogeneity was found (Chi^2^ = 4.65, *P* = 0.46, I^2^ = 0%). A significant association between acidemia and PVL was identified (OR, 1.87; 95% CI, 1.18 to 2.97).

### Low Apgar score and PVL

**Fig 4:**
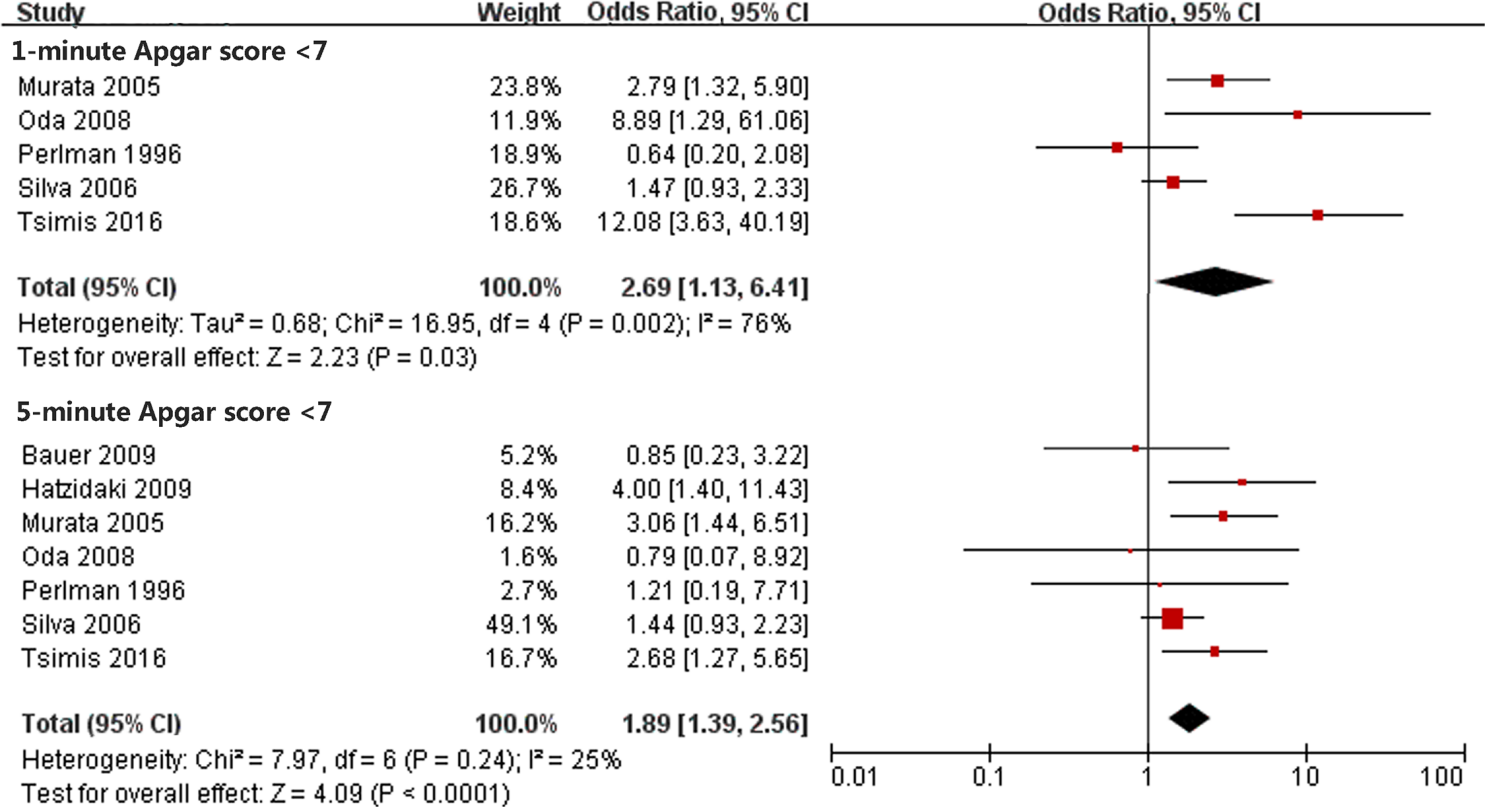
Forest plot of the odds ratio of the association between low Apgar score and PVL. A random-effect model was used to assess the association between low Apgar score at 1 minute and PVL. A fixed-effect model was used to assess the association between low Apgar score at 5 minutes and PVL. The pooled OR of low Apgar score is presented as a solid diamond at the bottom of the forest plot, the width of which represents the 95% CI.

Five of the studies used 1-minute Apgar score <7 and 7 studies analyzed 5-minute Apgar score <7 (Fig 4). Significant heterogeneity existed in the studies for 1-minute Apgar score <7 (Chi^2^ = 16.95, *P* = 0.002, I^2^ = 76%). Thus, a random-effect model was used. No significant heterogeneity was found in studies for 5-minute Apgar score <7 (Chi^2^ = 7.97, *P* = 0.24, I^2^ = 25%). Increased risk for PVL was found in infants with 1-minute Apgar score at birth <7 (OR 2.69; 95% CI, 1.13 to 6.41) or 5-minute (OR, 1.89; 95% CI, 1.39 to 2.56).

### Apnea and PVL

**Fig 5:**
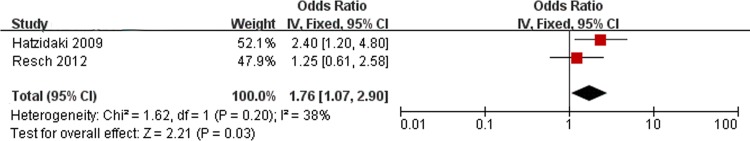
Fixed-effect model forest plot of the odds ratio of the association between apnea and PVL. The pooled OR of apnea is presented as a solid diamond at the bottom of the forest plot, the width of which represents the 95% CI.

Fig 5 presents the results of the combined ORs for apnea. There are 2 studies included in the meta-analysis. No significant heterogeneity was found (Chi^2^ = 1.62, *P* = 0.20, I^2^ = 38%). Analysis of the findings showed that apnea is associated with PVL (OR, 1.76; 95% CI, 1.07 to 2.90).

### RDS and PVL

**Fig 6:**
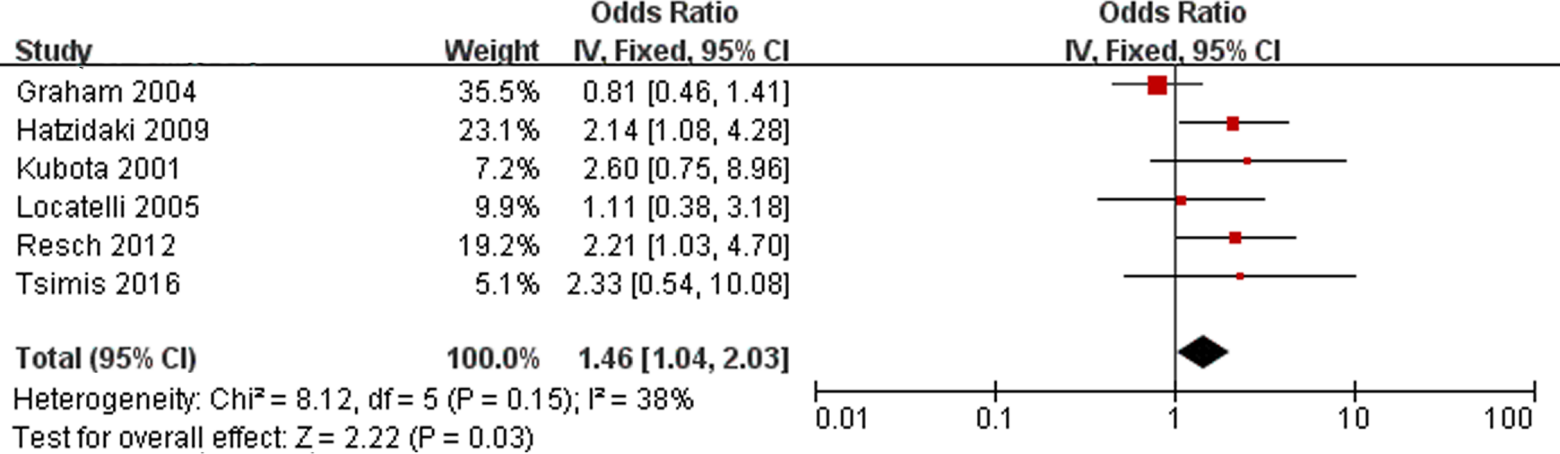
Fixed-effect model forest plot of the odds ratio of the association between RDS and PVL. The pooled OR of RDS is presented as a solid diamond at the bottom of the forest plot, the width of which represents the 95% CI.

Fig 6 shows the results of the combined ORs for RDS. There are 6 studies included in the meta-analysis that assessed RDS. No significant heterogeneity was found (Chi^2^ = 8.12, *P* = 0.15, I^2^ = 38%). Analysis of the findings showed that RDS is associated with PVL (OR, 1.46; 95% CI, 1.04 to 2.03).

### Seizures and PVL

**Fig 7:**
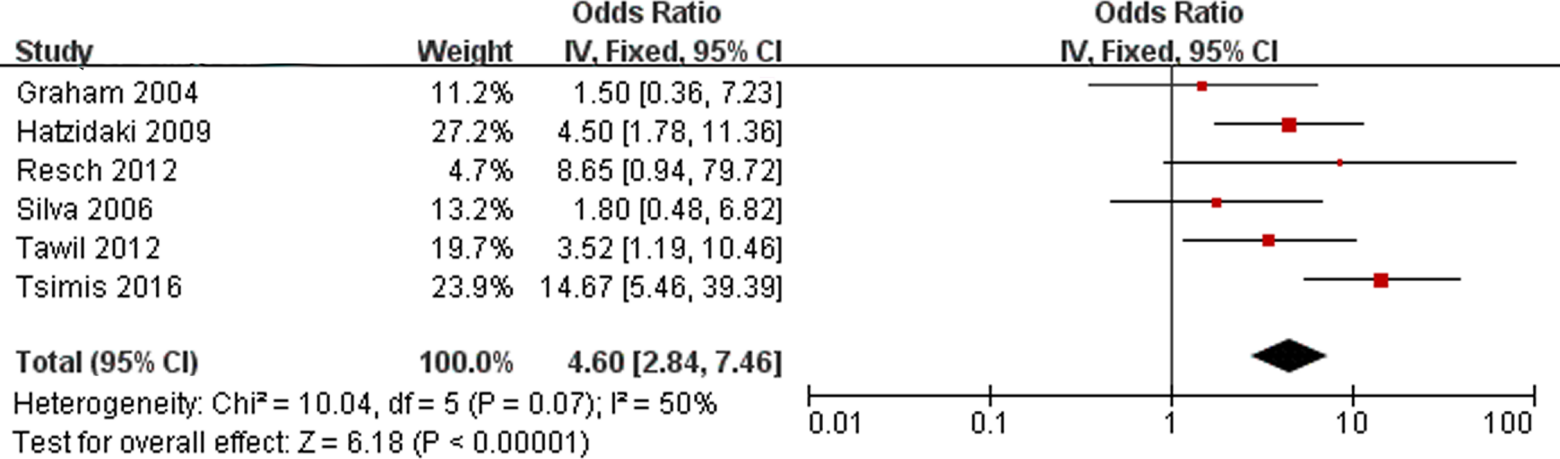
Fixed-effect model forest plot of the odds ratio of the association between seizures and PVL. The pooled OR of seizures is presented as a solid diamond at the bottom of the forest plot, the width of which represents the 95% CI.

Fig 7 shows the results of the combined ORs of neonatal seizures. There are 6 studies assessing seizures included in the meta-analysis. No significant heterogeneity was found (Chi^2^ = 10.04, *P* = 0.07, I^2^ = 50%). Analysis of the findings showed that seizures are associated with PVL (OR, 4.60; 95% CI, 2.84 to 7.46).

### Preeclampsia/eclampsia and PVL

**Fig 8:**
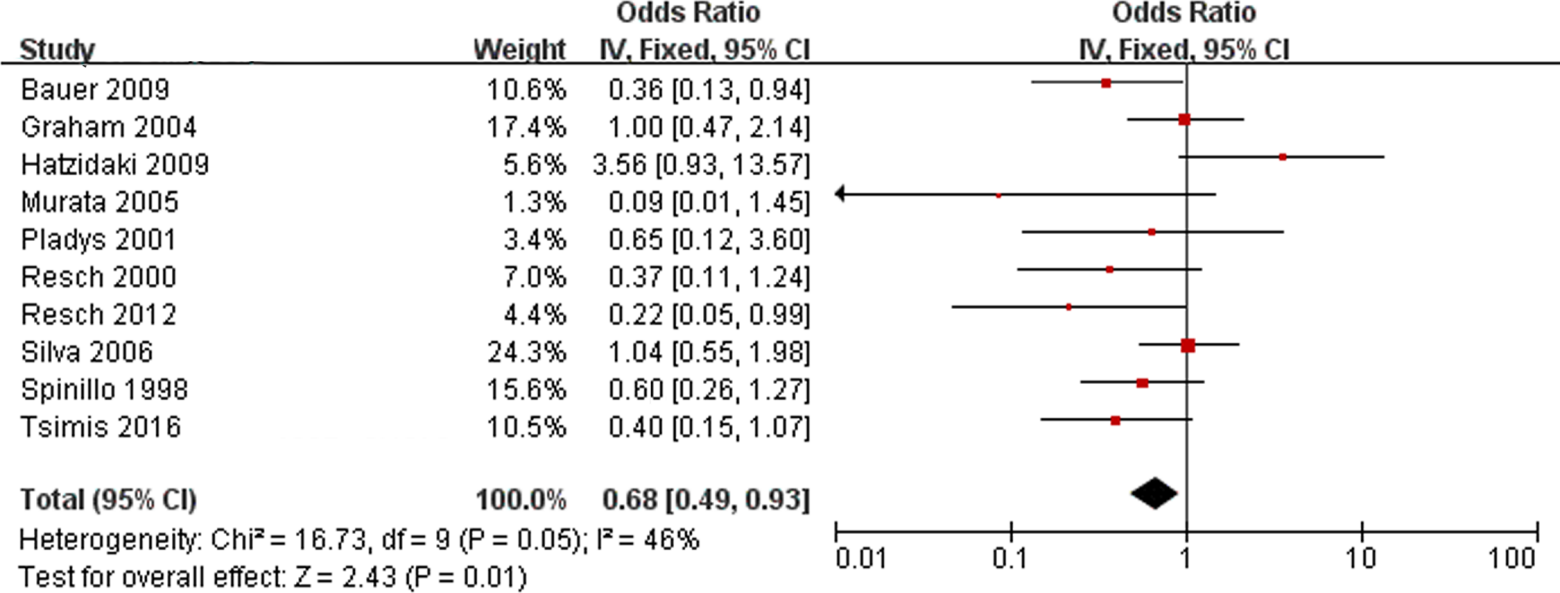
Fixed-effect model forest plot of the odds ratio of the association between preeclampsia/eclampsia and PVL. The pooled OR of preeclampsia/eclampsia is presented as a solid diamond at the bottom of the forest plot, the width of which represents the 95% CI.

Ten ORs of preeclampsia/eclampsia were pooled using the fixed-effects model (Fig 8). No significant heterogeneity was found (Chi^2^ = 16.73, *P* = 0.05, I^2^ = 46%). The pooled results indicated that maternal preeclampsia or eclampsia did not increase the risk of PVL in infants (OR, 0.68; 95% CI, 0.49 to 0.93).

### Publication bias

**Fig 9:**
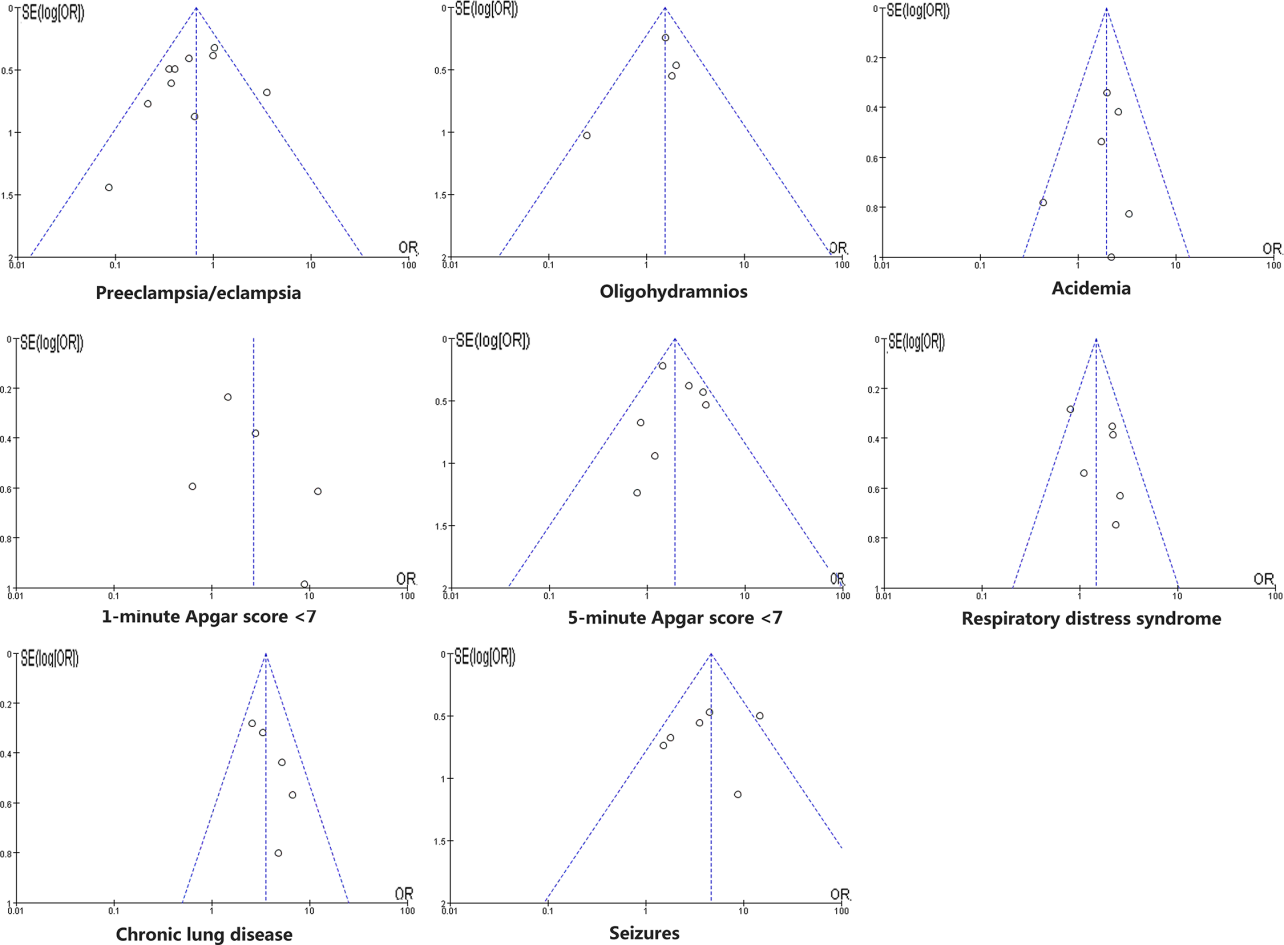
Funnel plot for publication bias test. The two oblique lines indicate the pseudo 95% confidence limits.

Publication bias was assessed in pooled analyses of more than two studies. A funnel plot was used for visual assessment of publication bias (Fig 9) and an adjusted Begg’s test was used to evaluate asymmetry and publication bias. The results of the Begg’s test indicated no evidence of publication bias (all *P* > 0.05).

## Discussion

Our meta-analysis included 15 studies and a total of 12,851 participants. The pooled results indicate that increased risk for PVL was associated with oligohydramnios in mothers (OR, 1.55; 95% CI, 1.05 to 2.30), acidemia (OR, 1.87; 95% CI, 1.18 to 2.97), 1-minute Apgar score <7 (OR 2.69; 95% CI, 1.13 to 6.41), 5-minute Apgar score <7 (OR, 1.89; 95% CI, 1.39 to 2.56), apnea (OR, 1.76; 95% CI, 1.07 to 2.90), RDS (OR, 1.46; 95% CI, 1.04 to 2.03), and seizures (OR, 4.60; 95% CI, 2.84 to 7.46) in preterm infants.

Previous epidemiological studies have shown that perinatal HI and inflammation are two major risk factors in the development of PVL [26]. Chorioamnionitis and preterm prelabor rupture of membranes (an important predictor of chorioamnionitis) have been identified as risk factors associated with PVL in preterm infants, as they lead to an exaggerated fetal inflammatory response syndrome [27–29]. Two previous meta-analyses have also demonstrated a relationship between chorioamnionitis and cerebral palsy—a major sequela of survivors of PVL [30, 31]. However, conclusions regarding the association between perinatal HI-related insults and PVL have been inconsistent. Our results demonstrate that certain HI-related factors increase the risk of PVL.

The fetus is highly susceptible to changes in maternal conditions, especially those associated with HI-related complications. Oligohydramnios may contribute to umbilical cord compression. In this meta-analysis, we found a significant difference between the relationship of oligohydramnios and the risk of PVL. However, oligohydramnios can occur secondary to some inflammatory events, such as preterm prelabor rupture of membranes [32]. Therefore, whether oligohydramnios can be considered an independent risk factor for the development of PVL remains to be discussed in further studies, as none of the studies included in this meta-analysis adjusted for inflammatory factors. In addition, in contrast to our expectation, we found that preeclampsia/eclampsia exerts a positive impact on the incidence of PVL. This protective effect may be associated with the continuous activation of the rennin–angiotensin system in the fetoplacental unit, which increases fetal cerebral circulation [33, 34]. Moreover, lower levels of exposure to intrauterine infections have been found in hypertensive mothers than in mothers with normal blood pressure [35].

Neonatal acidemia at birth is a crucial indicator for monitoring neurological outcomes after prenatal or perinatal hypoxic-ischemic events. It has been proposed that metabolic rather than respiratory acidosis is associated with neonatal neurologic deficits [36]. In most centers, umbilical cord pH between 7.0 and 7.2 is considered mild to moderate acidemia, and severe acidemia arises when cord pH decreases below 7.0. In addition, buffer base must be consumed before the drop in pH. As a result, base excess may be a more sensitive indicator for infants with significant acidemia. Low *et al*. [37] reported there is an increased risk of neonatal neurologic morbidity when the base excess falls to less than -12.0 mmol/l. Our results demonstrate an association between acidemia at birth and the development of PVL. An umbilical cord pH less than 7.2 or base excess less than -12.0 mmol/l may indicate PVL.

An Apgar score less than 7 is often considered to indicate fetal distress, and is more commonly associated with adverse neurologic outcomes [38]. Iliodromiti *et al*. [39] have reported that 5-minute Apgar score is more predictive of neonatal survival than that at 1 minute, and that lower Apgar score predict higher infant mortality. In contrast, although our results suggested that those preterm infants with Apgar score less than 7 at 1 minute or 5 minutes have an increased risk of PVL, the stronger relationship for PVL was found in preterm infants with low Apgar score at 1 minute rather than at 5 minutes. This difference may be due to the heterogeneity among the studies in the pooled analysis of PVL and low Apgar score at 1 minute. This heterogeneity is mainly due to the Tsimis *et al*. [13] study which only included infants with extremely low birth weight, whereas other studies included infants whose birth weights roughly ranged from 1,000 g to 1,500 g. The heterogeneity was eliminated when we excluded the Tsimis *et al*. [13] study from this pooled analysis. Previous studies have demonstrated that extremely low birth weight is associated with neurodevelopmental impairment and the development of PVL [40, 41]. Therefore, birth weight should be taken into consideration in future clinical studies of the association between low Apgar score and PVL.

Previous studies have shown that apnea [42], RDS [43], and seizures [44] can result in neurodevelopment impairment. These risk factors are therefore closely associated with the development of PVL, as indicated by our meta-analysis. In addition, hemodynamically significant patent ductus arteriosus (PDA) requiring surgical treatment is associated with large left-to-right ductal shunting, which may cause cerebral ischemia and hypoxia and then result in PVL [45]. However, our meta-analysis did not find a significant association between hemodynamically significant PDA and the development of PVL. The reason for this finding may be that most preterm infants with hemodynamically significant PDA undergo surgical closure of the PDA soon after birth. However, the timing of surgery to reduce the incidence of PVL in infants with hemodynamically significant PDA is still disputed [46]. Therefore, more studies should be performed to investigate the optimal timing of surgery to reduce the incidence of PVL in preterm infants.

To our knowledge, this is the first meta-analysis based on currently available studies to assess the association between perinatal hypoxic-ischemia and the development of PVL. However, this study has several limitations. First, all of the studies included in our analysis used medical records from one particular hospital or university rather than national health departments. As a result, our analysis lacks power. Second, all studies in our analysis were performed in the last 10 to 20 years. The diagnosis method used during this period was cranial ultrasound. However, in current clinical practice, the combination of magnetic resonance imaging and cranial ultrasound is used as the recognized diagnosis method to detect subtle lesions indicative of PVL. Thus, some cases of PVL may have been missed in our analysis. Third, none of the included studies adjusted for confounding variables such as necrotizing enterocolitis, which can also lead to systemic inflammation associated with subsequent development of PVL. Finally, the relatively small number of included studies resulted in the pooled results being underpowered.

Although this study was subject to a number of limitations, our meta-analysis identified perinatal hypoxic-ischemic risk factors for the development of PVL in preterm infants. Future large-scale prospective clinical studies are required to validate and extend these findings.

## Supporting information

S1 File PRISMA checklist. (DOC)
